# Comprehensive Genomic Landscape in Chinese Clear Cell Renal Cell Carcinoma Patients

**DOI:** 10.3389/fonc.2021.697219

**Published:** 2021-09-09

**Authors:** Jiwei Huang, Wen Cai, Biao Cai, Wen Kong, Wei Zhai, Jin Zhang, Yonghui Chen, Shiqing Chen, Yuezong Bai, Yiran Huang, Wei Xue

**Affiliations:** ^1^Department of Urology, Renji Hospital, School of Medicine, Shanghai Jiao Tong University, Shanghai, China; ^2^Department of Urology, The Second Affiliated Hospital, School of Medicine, Zhejiang University, Hangzhou, China; ^3^The Medical Department, 3D Medicines Inc., Shanghai, China

**Keywords:** clear cell renal cell carcinoma, next-generation sequencing, *VHL*, *PBRM1*, DDR genes

## Abstract

Clear cell renal cell carcinoma (ccRCC) is the most common subtype of renal cell carcinoma (RCC). The genomic landscape in Chinese ccRCC needs to be elucidated. Herein, we investigated the molecular features of Chinese ccRCC patients. Genomic profiling of DNA was performed through next-generation sequencing (NGS) in Chinese patients with ccRCC between January 2017 and March 2020. Clinical information including age, gender, and tumor histology was collected. Immunohistochemistry (IHC) staining for PD-L1 expression was performed using PD-L1 IHC 22C3 pharmDx assay or Ventana PD-L1 SP263 assay. Data analyses were performed using R 3.6.1. A total of 880 Chinese ccRCC patients who have undergone NGS were included in this study. The most common somatic alterations were detected in *VHL* (59.7%), *PBRM1* (18.0%), *SETD2* (12.2%), *BAP1* (10.2%), and *TP53* (9.4%). Compared with The Cancer Genome Atlas (TCGA) database, a higher mutation frequency of *VHL* (59.7% *vs.* 50.0%, p < 0.001) and *TP53* (9.4% *vs.* 3.5%, p < 0.001) and a lower mutation frequency of *PBRM1* (18.0% *vs.* 31.0%, p < 0.001) were found in the Chinese cohort. Of the 460 patients who were evaluated for PD-L1 expression, 139 (30.2%) had positive PD-L1 expression. The median tumor mutational burden (TMB) value was 4.5 muts/Mb (range, 0–46.0). Five (0.7%) patients were identified as microsatellite instability-high (MSI-H). Furthermore, 52 (5.9%) patients were identified to carry pathogenic or likely pathogenic germline mutations in 22 cancer predisposition genes. This is the first large-scale comprehensive genomic analysis for Chinese ccRCC patients, and these results might provide a better understanding of molecular features in Chinese ccRCC patients, which can lead to an improvement in the personalized treatment for these patients.

## Introduction

Clear cell renal cell carcinoma (ccRCC) is the most common subtype of renal cell carcinoma (RCC), accounting for 70%–85% of individuals (1–3). Such cancer subtype is almost uniformly lethal and represents critical distinction. In view of its lack of sensitivity to radiation and chemotherapy, many efforts have been made to explore biomarker–biomarker-oriented therapy (4). Some targeted therapeutic agents targeting vascular endothelial growth factor (VEGF) signaling, such as sunitinib and pazopanib, have greatly improved the prognosis of ccRCC patients. Additionally, immune checkpoint inhibitor alone or combined strategies were also identified as a promising therapeutic target for those patients (5, 6). Revealing comprehensive genomic features is of great importance for understanding ccRCC and developing new therapeutic lines for patients with ccRCC (7).

Some previous studies have reported the genomic landscape of ccRCC (8). For example, The Cancer Genome Atlas (TCGA) has conducted comprehensive molecular characterizations in ccRCC, including alterations in genes controlling cellular oxygen sensing (for example, *VHL*) and the maintenance of chromatin states (for example, *PBRM1*) (9). However, most of the researches were conducted on patients from Western countries or focused on the prognostic value of gene alteration (10, 11). The genomic landscape in Chinese ccRCC still needs to be elucidated. Recently, one study explored the somatic mutations in 26 Chinese patients with primary RCC by whole exome sequencing and only included 15 ccRCC patients (12). It is necessary to systematically study the gene alteration, as well as their relationship with immune-related biomarkers in a large Chinese patient.

Recently, next-generation sequencing (NGS) had be applied in clinical practice and in changing the clinical management of ccRCC. In the present study, we investigated the molecular features of Chinese ccRCC patients by NGS. This study aimed to systematically study the genomic landscape in Chinese ccRCC, which will plays an increasingly important role in precision medicine of ccRCC.

## Methods

### Clinical Specimens

We retrospectively analyzed genomic profiling of DNA performed through NGS in Chinese patients with ccRCC between January 2017 and March 2020. The NGS testing of tumor DNA in formalin-fixed paraffin-embedded (FFPE) samples was performed by customized NGS panel targeting 381 or 733 cancer-related genes in a Clinical Laboratory Improvement Amendments-certified and College of American Pathologists-accredited laboratory (3DMedcines, Inc., China). Notably, all the genes in a 381-gene panel were included in a 733-gene panel. The clinicopathologic characteristics including age and sex were collected. All participated patients provided written consent, and the study was approved by the ethics committee of Renji Hospital.

### DNA Sequencing

FFPE tissue sections were evaluated for tumor cell content using hematoxylin and eosin (H&E) staining. Only samples with a tumor content of ≥20% were eligible for subsequent analyses. DNA extracts (30–200 ng) were sheared to 250-bp fragments using an S220 focused ultrasonicator (Covaris). Libraries were prepared using the KAPA Hyper Prep Kit (KAPA Biosystems) following the manufacturer’s protocol. For targeted capture, indexed libraries were subjected to probe-based hybridization with a customized NGS panel by following manufacturer’s instruction. The concentration and fragment size distribution of the final library were determined using a Qubit 3.0 fluorometer (Thermo Fisher Scientific) and a LabChip GX Touch HT Analyzer (PerkinElmer), respectively. The captured libraries were loaded onto a NovaSeq 6000 platform (Illumina) for 100-bp paired-end sequencing with a mean sequencing depth of 1,500.

As described previously (13), raw data of tissue samples were mapped to the reference human genome hg19 using the Burrows–Wheeler Aligner (v0.7.12). Somatic single-nucleotide variants (SNVs) were detected using MuTect (v1.1.7) (https://github.com/broadinstitute/mutect); and somatic insertions and deletions (indels) were detected using Pindel (v0.2.5a8) (http://gmt.genome.wustl.edu/packages/pindel) with default parameters. Single-nucleotide polymorphisms (SNPs) and indels were annotated by ANNOVAR against the following databases: dbSNP (v138), 1000 Genomes, and ESP6500 (population frequency > 0.015). Only missense, stopgain, frameshift, and non-frameshift indel mutations were retained. Tumor mutational burden (TMB) was defined as somatic mutation counts in coding regions per megabase of genome examined. SNVs included both synonymous and non-synonymous mutations, as well as stopgain, stoploss, and splicing variants.

### PD-L1 Staining

Immunohistochemistry (IHC) staining for PD-L1 expression was performed using PD-L1 IHC 22C3 pharmDx assay or Ventana PD-L1 SP263 assay. PD-L1 positive was defined as tumor proportion score (TPS) ≥1% (**Supplementary Figure 1**).

### Statistical Analysis

The demographic characteristics of patients were compared *via* the chi-square (χ^2^) test or t-test. All p-values presented were two-sided, and associations were considered significant if the p-value was less than 0.05. Statistical analyses were performed using R version 3.6.1 (R Foundation for Statistical Computing) and GraphPad Prism v6 (GraphPad, La Jolla, CA, USA).

## Results

A total of 880 Chinese ccRCC patients who have undergone NGS with a 381-gene panel (n = 744) or a 733-gene panel (n = 136) were included in this study, including 620 (70.5%) male and 260 (29.5%) female patients (**Table 1**). The median age was 55 (range, 14–87). Importantly, PD-L1 is associated with improved overall response rates (ORRs) and prolonged progression-free survival (PFS) in metastatic RCC (mRCC) patients receiving immunotherapy (14). Thus, IHC staining for PD-L1 expression was performed in 460 patients, using PD-L1 IHC 22C3 pharmDx assay or Ventana PD-L1 SP263 assay.

**Table 1:** Clinicopathologic features.

Characteristics		
Sample, n		880
Age, median (range)		55 (14–86)
Sex, n (%)		
Male		620 (70.5%)
Female		260 (29.5%)
PD-L1, n (%)		
Negative		321 (69.8%)
Positive	≥1%	139 (30.2%)
	≥10%	70 (15.2%)
	≥50%	30 (6.5%)
MSI		
MSI-H		5 (0.7%)
MSS		665 (99.3%)
TMB, median (range)		4.5 (0–46.0)

MSI-H, microsatellite instability-high; MSS, microsatellite stable; TMB, tumor mutational burden.

**Figure 1:**
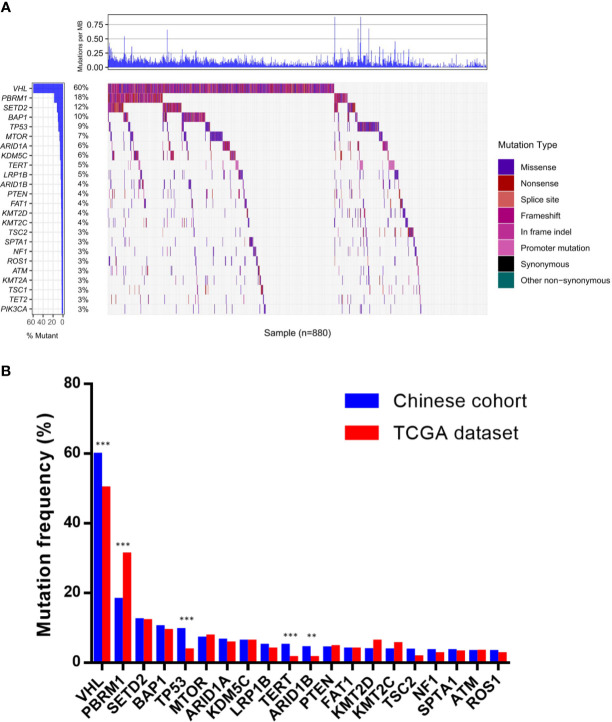
Molecular features of Chinese clear cell renal carcinoma. **(A)** The somatic mutation landscape in Chinese clear cell renal carcinoma patients. **(B)** Discrepancies of mutation frequency between Chinese cohort and TCGA dataset. **p < 0.01, ***p < 0.001. TCGA, The Cancer Genome Atlas.

In Chinese ccRCC patients, 95.8% harbored at least one pathogenic mutation. The most common somatic alterations were detected in *VHL* (59.7%), *PBRM1* (18.0%), *SETD2* (12.2%), *BAP1* (10.2%), and *TP53* (9.4%) (**Figure 1A**). The gene mutational landscape of Chinese ccRCC patients was similar with that of TCGA database; however, a higher mutation frequency of *VHL* (59.7% *vs.* 50.0%, p < 0.001) and *TP53* (9.4% *vs.* 3.5%, p < 0.001) and a lower mutation frequency of *PBRM1* (18.0% *vs.* 31.0%, p < 0.001) were found in the Chinese cohort (**Figure 1B**).

**Figure 2:**
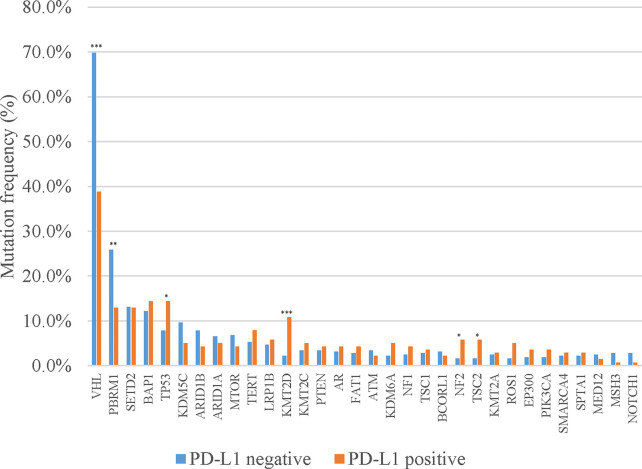
Discrepancies of mutation frequency between PD-L1-positive and PD-L1-negative patients. *p < 0.05, **p < 0.01, ***p < 0.001.

**Figure 3:**
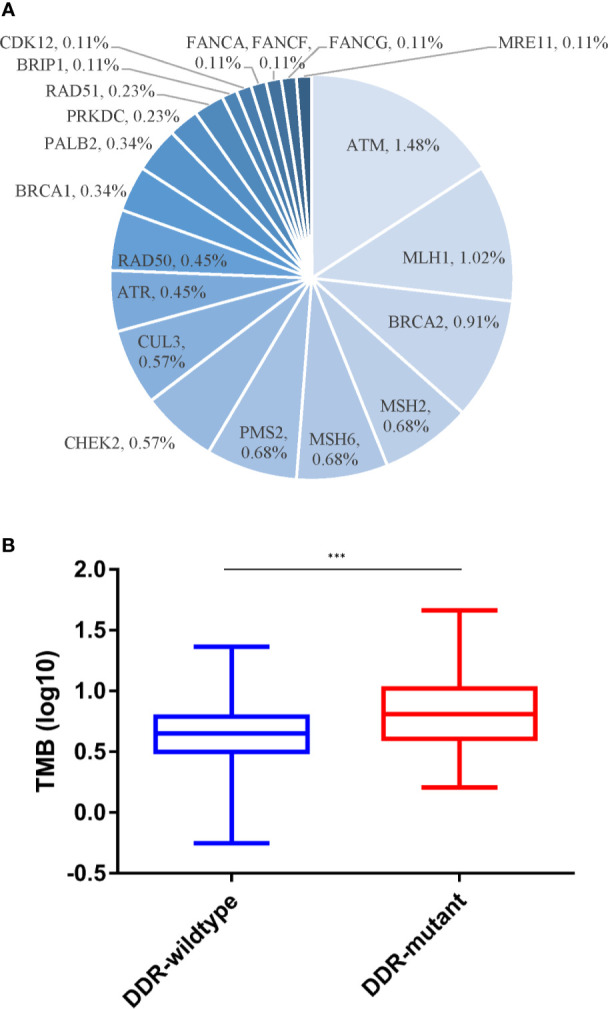
Somatic DDR alterations and TMB. **(A)** Distribution of DDR gene mutations. **(B)** Discrepancies of TMB between DDR-mutant and DDR-wild type group. DDR, DNA damage repair; TMB, tumor mutational burden. ***p < 0.001.

Of the 460 patients who were evaluated for PD-L1 expression, 139 (30.2%) had positive PD-L1 expression (TPS ≥1%). In addition, a significantly lower mutation frequency of *VHL* and a higher mutation frequency of *PBRM1* were observed among patients with PD-L1-positive tumors, compared with those with PD-L1-negative tumors (*VHL*: 38.8% *vs.* 69.8%, p < 0.001; *PBRM1*: 25.9% *vs.* 12.9%, p = 0.002) (**Figure 2**). We also evaluated the TMB in Chinese ccRCC patients. The median TMB value was 4.5 muts/Mb (range, 0–46.0). Previous studies indicated that DNA damage repair (DDR) gene mutations were associated with TMB. We also found somatic mutations in several DDR genes, including *ATM* (1.5%), *MLH1* (1.0%), and *BRCA2* (0.9%). The patients with DDR mutations presented higher TMB (median TMB = 6.5 *vs.* 4.3 muts/Mb, p < 0.001) (**Figure 3**). Five (0.7%) patients were identified as microsatellite instability-high (MSI-H).

**Figure 4:**
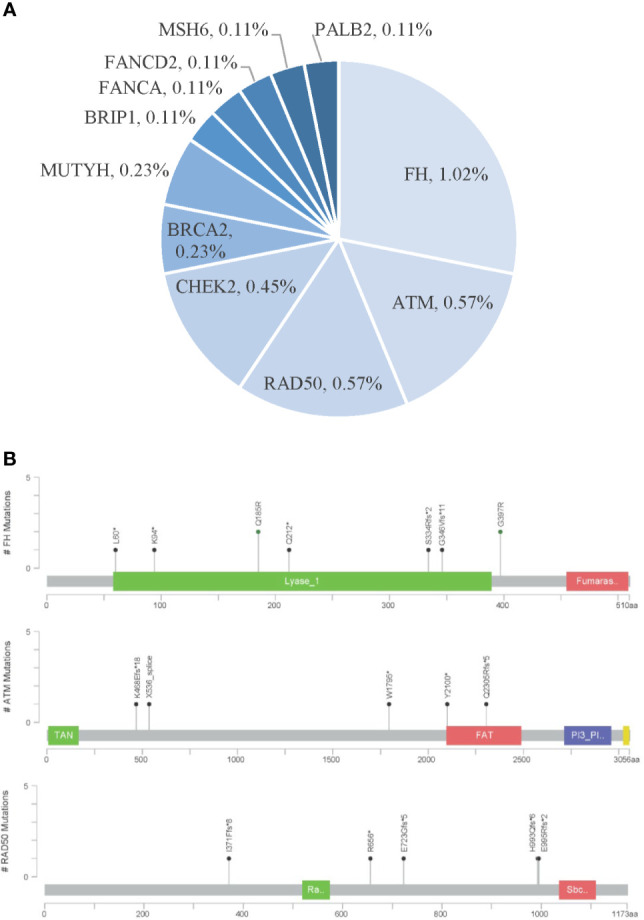
Pathogenic germline variants in Chinese clear cell renal carcinoma. **(A)** Distribution of pathogenic germline mutations. **(B)** Locations of pathogenic germline mutations in the top three frequent germline mutant genes.

Furthermore, 52 (5.9%) patients were identified to carry pathogenic or likely pathogenic germline mutations in 22 cancer predisposition genes. The frequent germline mutant genes in Chinese ccRCC patients included *FH* (1.0%), *ATM* (0.57%), *RAD50* (0.57%), *CHEK2* (0.45%), *FLCN* (0.45%), and *VHL* (0.45%) (**Figure 4**).

## Discussion

To the best of our knowledge, this is the first large-scale comprehensive genomic analysis of Chinese ccRCC patients. We revealed the molecular features of Chinese ccRCC patients; and we found mutation frequencies of some key driver genes, such as *VHL* and *PBRM1*, in ccRCC, which was different from those of the TCGA dataset. In addition, our results also showed the relationship between PD-L1 expression and *VHL* or *PBRM1*, and the association between DDR gene mutations and TMB.

In both TCGA dataset and our cohort, *VHL* and *PBRM1* were the most commonly altered genes. Compared with TCGA database, a higher mutation frequency of VHL (59.7% *vs.* 50.0%, p < 0.001) and TP53 (9.4% *vs.* 3.5%, p < 0.001) and a lower mutation frequency of PBRM1 (18.0% *vs.* 31.0%, p < 0.001) were found in the Chinese cohort. Such difference might result from different ethnicity (15, 16). In a previous study (12), Wang et al. reported that the mutation frequency of *VHL* was 67% in ccRCC, which is slightly higher than that of our cohort (59.7%). And the mutation frequency of *PBRM1* was only 7% in that study (12), lower than our data (18.0%). Such difference may be due to the small sample sizes in Wang’s study.

*VHL* is a tumor suppressor gene and plays a key role in cellular oxygen sensing and the tumorigenesis of ccRCC. Previous studies demonstrated that inactivation of *VHL* was not significantly associated with anti-VEGF receptor (anti-VEGFR) inhibitors (17, 18); however, it might predict the efficacy of HIF-2 inhibitors (19). In our study, PD-L1-positive expression was associated with a lower *VHL* mutation frequency, which indicates that most *VHL*-mutated ccRCC patients might not receive benefit from anti-PD-1/L1 inhibitors combined with anti-VEGFR inhibitors. *PBRM1* encodes a subunit of SWI/SNF chromatin-remodeling complexes; and truncating mutations in *PBRM1* was demonstrated to be associated with clinical benefit from anti-PD-1/L1 inhibitors (20). Furthermore, we showed the distribution of PD-L1 expression and TMB in Chinese ccRCC, and PD-L1 expression and TMB could predict the efficacy of anti-PD-1/L1 inhibitors across multi-type tumors (21). Several studies indicated that DDR alterations were associated with high TMB, also predicting the clinical activity of anti-PD-1/L1 inhibitors (22, 23). For the first time, we reported the relationship between DDR mutations and TMB in ccRCC, which may provide more predictive biomarkers for anti-PD-1/L1 inhibitors in ccRCC.

There are several limitations to this study. First, this present work was a retrospective research with 880 cases that could not avoid selection bias. Second, except for age and sex, clinicohistological characteristics such as cancer subtype, treatment history, and survival outcomes of these cases are missed. Thus, the effect of the biomarkers on treatment decisions and its correlation with survival outcomes need to be further confirmed in further studies. With development of the liquid biopsy technique, some hematochemical biomarkers, such as circulating tumor cells and circulating tumor DNA (ctDNA), have been applied widely in various urinary tumor management (24–28). CtDNA status and cfDNA fragment size are clinically used as biomarkers for prognosis and disease monitoring in RCC (29, 30). While studies of ctDNA in RCC are still in their infancy, larger-scale prospective studies with complete clinical information should be carried out to further validate such findings.

In conclusion, we first reported the large-scale comprehensive genomic features of Chinese ccRCC patients, as well as the relationship between immunotherapy biomarkers and gene alteration. These results might provide a better understanding of molecular features in Chinese ccRCC patients, which might promote an improvement in the personalized treatment for these patients.

## Data Availability Statement

The datasets presented in this article are not readily available because making data publicly available would compromise patient confidentiality, and sequencing data contain sequencing algorithm and other core trade information of 3D Medicines Inc. Requests to access the datasets should be directed to the corresponding author YH.

## Ethics Statement

The studies involving human participants were reviewed and approved by the Ethics Committee of Renji Hospital. Written informed consent to participate in this study was provided by the participants’ legal guardian/next of kin.

## Author Contributions

WX, YH, and JH contributed to the conception and design of the study. JH, WC, BC, WK, WZ, JZ, YC, SC, and YB organized the database. SC and YB performed the statistical analysis. JH, WC, and BC wrote the first draft of the manuscript. WK, WZ, JZ, YC, and SC wrote sections of the manuscript. All authors contributed to the article and approved the submitted version.

## Funding

This research was supported by Shanghai Science and Technology Commission Research Project (18ZR1423200), the Incubating Program for Clinical Research and Innovation of Renji Hospital (PYXJS16-008, PYIII20-07), Shanghai Natural Science fund of Shanghai (21ZR1438900), and Basic Oncology Research Program from Bethune Charitable Foundation (BCF-NH-ZL-20201119-024).

## Conflict of Interest

Authors SC and YB were employed by company 3D Medicines Inc.

The remaining authors declare that the research was conducted in the absence of any commercial or financial relationships that could be construed as a potential conflict of interest.

## Publisher’s Note

All claims expressed in this article are solely those of the authors and do not necessarily represent those of their affiliated organizations, or those of the publisher, the editors and the reviewers. Any product that may be evaluated in this article, or claim that may be made by its manufacturer, is not guaranteed or endorsed by the publisher.
