# Supplementary material for: Comprehensive Genomic Landscape in Chinese Clear Cell Renal Cell Carcinoma Patients
Source: Front Oncol. 2021 Sep 9;11:697219. doi: 10.3389/fonc.2021.697219 (PMC8459629; doi:10.3389/fonc.2021.697219)
Supplement: Supplementary file 1 [file DataSheet_1.docx]

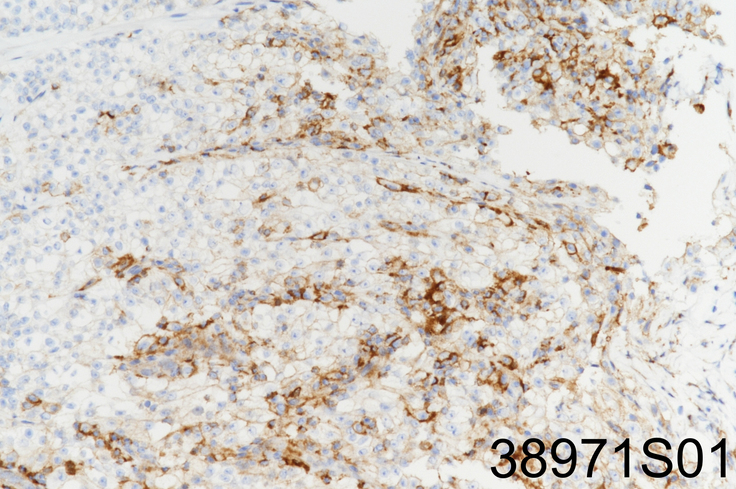


**Supplementary figure 1. IHC staining for PD-L1 expression.** Ventana PD-L1 SP263 assay was used and the tumor positive score (TPS) ≥ 1 %.
